# The influence of non-native language proficiency on speech perception performance

**DOI:** 10.3389/fpsyg.2014.00651

**Published:** 2014-07-02

**Authors:** Lisa Kilman, Adriana Zekveld, Mathias Hällgren, Jerker Rönnberg

**Affiliations:** ^1^Department of Behavioural Sciences and Learning, Linköping UniversityLinköping, Sweden; ^2^Linnaeus Centre HEAD, Swedish Institute for Disability Research, Departement of Behavioural Sciences and Learning, Linköping UniversityLinköping, Sweden; ^3^Department of Audiology/ENT, EMGO Institute for Health and Care Research, VU University Medical CenterAmsterdam, Netherlands; ^4^Department of Otorhinolaryngology, Section of Audiology, Linköping University HospitalLinköping, Sweden

**Keywords:** English proficiency, native, non-native, speech perception, informational masking, energetic masking, working memory

## Abstract

The present study examined to what extent proficiency in a non-native language influences speech perception in noise. We explored how English proficiency affected native (Swedish) and non-native (English) speech perception in four speech reception threshold (SRT) conditions, including two energetic (stationary, fluctuating noise) and two informational (two-talker babble Swedish, two-talker babble English) maskers. Twenty-three normal-hearing native Swedish listeners participated, age between 28 and 64 years. The participants also performed standardized tests in English proficiency, non-verbal reasoning and working memory capacity. Our approach with focus on proficiency and the assessment of external as well as internal, listener-related factors allowed us to examine which variables explained intra- and interindividual differences in native and non-native speech perception performance. The main result was that in the non-native target, the level of English proficiency is a decisive factor for speech intelligibility in noise. High English proficiency improved performance in all four conditions when the target language was English. The informational maskers were interfering more with perception than energetic maskers, specifically in the non-native target. The study also confirmed that the SRT’s were better when target language was native compared to non-native.

## INTRODUCTION

Speech comprehension in noisy conditions and in a non-native language is a challenging process that requires full attention of the listener. To perceive words as meaningful in such situations involves perceptual, linguistic, and cognitive abilities as well as knowledge of the current language. Proficiency in a non-native language might even be more important when the speech signal is degraded as compared to clear speech. The relevance of proficiency has been claimed in previous research (van Wijngaarden et al., 2002; Weiss and Dempsey, 2008; Van Engen, 2010; Brouwer et al., 2012), although its plausible role in non-native speech perception has not, to our knowledge, been the main focus in relevant research so far. This study provides a new approach by shifting the focus and by an extended design that assesses the variables that possibly explain interindividual differences in non-native speech perception. The general aim of the current study is to analyze the contribution of non-native language proficiency in native and non-native speech perception in different types of interfering maskers.

Assessment of speech perception in a non-native language entails a design that acknowledges the complexity of the perception process by addressing both external and internal (listener-related) factors that might influence perception. Regarding the external factors affecting non-native speech perception, it is useful to consider the different characteristics of energetic and informational maskers (Brungart, 2001). Energetic masking refers to the spectro-temporal overlap between the target speech and interfering maskers such as multi-talker babble, fluctuating noise, or stationary noise. When the masker has a fluctuating amplitude, limited parts of the target signal are audible in the dips of the masker (Festen and Plomp, 1990; Cooke, 2006). The term “informational masking” is used for any masking effects that are not caused by energetic masking. This term is usually applied for meaningful words or sentences that can be understood by the listener and are therefore likely competing with the target signal. Accordingly, informational maskers result in attention distraction, semantic intrusion, and increased cognitive load (Mattys et al., 2009, 2012). The type of masker affects the degree to which explicit processes are required to perceive the speech (Rönnberg et al., 2010, 2013). If the target and the masker are both in the native language, the parallel speech signals are likely competing with each other in a more interfering way than when a non-native or unfamiliar speech masker is used that might be easier to suppress (). Several studies have confirmed the effects of the language of the masker speech on the listeners’ recognition of the native target language (Rhebergen et al., 2005; Garcia Lecumberri and Cooke, 2006; Van Engen and Bradlow, 2007; Calandruccio et al., 2010). The findings support the relative release of masking when the competing speech is in an unfamiliar or foreign language compared to maskers in the listeners’ native language. In the study of Van Engen and Bradlow (2007), native English participants were better able to perceive English target speech masked by two-talker Mandarin babble than when two-talker English babble masked the speech. Van Engen and Bradlow (2007) concluded that, under certain conditions, the language of the interfering speech can affect the intelligibility of the target speech. Brouwer et al. (2012) formalized this suggestion by formulation of the “linguistic-similarity hypothesis.” This hypothesis states that the more similar the target and the masker are, the more difficult it is to keep apart the two streams efficiently. Brouwer et al. (2012) tested this hypothesis in three experiments. Native monolingual American English participants and native Dutch-English bilingual (i.e., highly proficient in English) participants were tested. The interfering speech consisted of two-talker babble in English and Dutch as well as semantically anomalous two-talker babble in English and Dutch, in each of the three experiments. In the first experiment, the target speech was American-English. The monolingual American-English listeners in the experiment had better speech perception for the unfamiliar Dutch masker as compared to the English masker (i.e., they received a release from masking for the non-native masker speech). In the second experiment, again with American-English as target speech, the native Dutch bilingual listeners received a release from masking when the competing speech was different (Dutch) from the target speech (English) even though the masker was in their native and the target was in their non-native language. However, this release from masking was smaller than that observed for the American-English monolingual listeners. In the third experiment, another group of Dutch bilingual (i.e., highly proficient in English) listeners perceived Dutch target speech. They received a release from masking for the English as opposed to the Dutch background babble. Brouwer et al. (2012) concluded that the results support the target-masker linguistic-similarity hypothesis, which determines the accuracy of speech-in speech recognition.

Van Engen (2010) observed similar effects when exploring how monolingual native English participants and bilingual speakers of Mandarin, with English as second language, recognized English target speech in English or Mandarin two-talker babble. Both groups showed greater difficulty in English babble than in Mandarin babble. However, the native Mandarin-speaking participants experienced relatively more interference from Mandarin babble than the monolingual English participants. The large interference from the English masker on the English target suggests that the linguistic similarity between the masker and the target speech made them difficult to separate. The informational masking effect of the Mandarin babble was larger for the Mandarin speakers because they were more proficient in this language.

In terms of internal, listener-related factors affecting non-native speech perception, the amount of available cognitive resources is a relevant factor. For speech perception performance in challenging conditions, working memory capacity has proven to be an important predictor (e.g., Rönnberg et al., 2008; Kramer et al., 2009). In fact, working memory is essential when language in any form is perceived (Baddeley, 2003) and is the operational ability to process, store and form conclusions about information (Hannon and Daneman, 2001). Yet this cognitive storage and processing capacity is limited (Baddeley, 2000) and differs between individuals (Daneman and Carpenter, 1980; Just and Carpenter, 1992). In suboptimum conditions, working memory capacity can be taxed for various reasons like a poor signal or unfocused attention (Shinn-Cunningham, 2008). The relative reliance on working memory processes depends on the degree to which the quality of the auditory signal allows easy speech perception. Signal aspects influence the ability to use stimulus-driven processes to separate acoustic components in the surrounding area (Bregman, 1990). Knowledge-driven processes contribute to these stimulus-driven processes in understanding speech in the presence of other sound sources (Plomp, 2002; Rönnberg et al., 2010). For example, knowledge-driven processes take advantage of previous knowledge in the interpretation of the incoming signals (Zekveld et al., 2011). If the reliance on knowledge-driven processes increases, the relationship between working memory capacity and speech perception performance becomes stronger (Zekveld et al., 2011). Decoding a non-native language in a noisy condition probably requires more knowledge-driven processes than native language perception.

To allow assessment of the role of working memory in non-native speech perception, we included the Reading Span test as measure of working memory capacity in the current study (Daneman and Carpenter, 1980; Rönnberg et al., 1989). The Reading Span Test is a dual task and requires parallel semantic processing and memory storage. It is frequently used and is a robust predictor of individual performance in speech-in-noise tasks (e.g., Foo et al., 2007; Rudner et al., 2009; Zekveld et al., 2011). We included both a Swedish (native) and an English (non-native) version of the test in order to assess whether the performance on the tests would differ, and whether any difference would relate to non-native language proficiency.

Besides working memory, another internal, listener-related factor influencing speech perception is non-native language proficiency. Examination of the relationship between proficiency and speech recognition in noise in non-native and native speech maskers, provides more insight into the degree to which this ability explains interindividual differences in perception. Many studies have either assessed the role of language proficiency in an all-or-nothing fashion by comparing monolingual versus bilingual listeners (e.g., Brouwer et al., 2012), or applied subjective self-rating scales to assess proficiency in the non-native language (van Wijngaarden et al., 2002; Weiss and Dempsey, 2008; Broersma and Scharenborg, 2010; Cooke et al., 2010; Brouwer et al., 2012). A disadvantage of using self-rating scales is that they have their own intrinsic subjectivity and are not the most reliable measure of proficiency. In the current study, we aimed to assess non-native linguistic proficiency using an objective, continuous measure. Therefore, we applied a standardized proficiency test that enables us to examine the degree to which non-native language proficiency affects speech perception in different noise and speech maskers.

To facilitate the detection of internal, listener-related factors on non-native speech perception, the age-range of the participants in the current study was relatively wide as compared to previous studies that commonly included young students as participants (e.g., Bradlow and Alexander, 2007; Cooke et al., 2008; Weiss and Dempsey, 2008; Mattys et al., 2010; Van Engen, 2010; Brouwer et al., 2012). Young, highly educated listeners usually have relatively homogenous scores and are not representative for the population as a whole. Including a group more representative of the general population with regard to age and education resulted in more between-subjects variance on the independent and dependent variables. This provided a better opportunity to find relations between the variables and increased the generalizability of the results. Note that pure-tone hearing thresholds of the listeners were measured to ensure that hearing acuity of all listeners was within a normal range.

We also included Raven standard progressive matrices (Carpenter et al., 1990) as a listener-related factor. The Raven standard progressive matrices were included to investigate whether a general measure of non-verbal intelligence would be associated with speech perception in difficult conditions. We examined the relation between these variables and speech perception in noise in Swedish and English.

For assessing the external factors that might affect interindividual differences in native and non-native speech perception, we applied Speech Reception Threshold (SRT) tests. SRT tests provide sensitive estimates of the listeners’ ability to perceive speech in background signals. Due to the adaptive method applied in the test, there is no risk for ceiling or floor effects in the listeners’ performance (Plomp and Mimpen, 1979). The aim of the current study was to examine the influence of energetic and informational masking on Swedish (native) and English (non-native) speech perception under different masker types. Four types of maskers were used; stationary and fluctuating noise that mainly result in energetic masking and two-talker babble in Swedish and two-talker babble in English that also result in informational masking.

The approach adopted in the present study differs from previous studies regarding several important aspects. The objective assessment of non-native language proficiency and the extended within-subjects design applied in the current study allowed us to sensitively assess the variables explaining interindividual differences in native and non-native speech perception performance. As described above, the complex interactions between relevant factors in non-native speech perception require the assessment of both external and internal, listener-related factors. The current study addressed the roles of (a) non-native proficiency as measured on a continuous, objective scale, (b) working memory capacity as assessed in the native and non-native language while (c) using adaptive SRT tests with both energetic and informational masker signals. The listeners had a relatively wide age-range while normal hearing was ensured by assessment of the audiogram. These factors extend the design of previous studies in this field of research (e.g., Garcia Lecumberri and Cooke, 2006; Bradlow and Alexander, 2007; Cooke et al., 2008; Weiss and Dempsey, 2008; Broersma and Scharenborg, 2010; Van Engen, 2010; Brouwer et al., 2012).

We expected that high proficiency, large working memory capacity, and high Raven scores would be associated with better speech perception, especially in the most difficult conditions, which is in line with the ELU-model (Rönnberg, 2003; Rönnberg et al., 2008, 2013). We predicted that the SRTs would be lower (better) when target speech is in Swedish than when target speech is in English. Due to the large masking effect of speech maskers as observed previously (Brungart et al., 2001; Van Engen and Bradlow, 2007; Calandruccio et al., 2010), we expected worse speech perception for the informational maskers compared to the energetic maskers. We also expected larger interference effects for the linguistically similar maskers (e.g., English maskers for English target speech) than for the dissimilar maskers (Brouwer et al., 2012).

## MATERIALS AND METHODS

### PARTICIPANTS

Twenty-three native Swedish listeners participated, including 13 females and 10 males. The ages ranged from 28–64 years (*M* = 49.5, SD = 9.8). Participants were recruited from different workplaces. The participants filled out a questionnaire in which they answered several questions about their knowledge of, training in and use of English. Their years of education ranged from 11–21 years (*M* = 15.8). All had learned English, starting from the third, fourth, or fifth grade in primary school and considered English as their second language. However, the frequency with which they actually used English in daily life varied from every day to never. Pure-tone hearing thresholds of the participants were measured to ensure the thresholds of both ears were ≤20 dB HL at the octave frequencies between 125 and 4000 Hz. The participants had pure tone hearing thresholds of maximal 25 dB HL between 125 and 2000 Hz and of maximal 35 dB HL at 4000 Hz. One participant had a threshold of 45 dB HL at 4000 Hz in one ear. All participants provided written informed consent in accordance with the Ethics Committee. They received a small gift for taking part.

### EXPERIMENTAL TEST AND STIMULI

The SRT test was applied to measure sentence intelligibility under the influence of noise (Plomp and Mimpen, 1979). In the SRT tests we presented Swedish or English target speech.

*Speech materials* – In the SRT tests, we either applied Swedish (Hällgren et al., 2006) or American English HINT (Nilsson et al., 1994) sentences. The HINT material – in both languages – consists of short everyday sentences, which have been judged to be natural by native speakers. The sentence materials are grouped into 25 phonemically balanced lists of 10 sentences each. The sentences are recorded by male speakers. Noise onset was 3 s before speech onset and noise offset was 1 s after speech offset. Participants performed eight test conditions; target speech in Swedish or English orthogonally combined with four types of masker: stationary noise, fluctuating noise, two-talker babble in Swedish, and two-talker babble in English respectively (see description below). Each condition contained 20 sentences and every new condition involved practice; the first with 10 sentences and the following with 5 sentences each. The order of conditions was counterbalanced across participants and each sentence was used only once.

Speech was presented at a fixed level of 65 dB SPL. The participants listened to the sentence in noise and responded orally by repeating the sentence. The experimenter compared the response with the actual sentence and if each word was accurate, the noise level for the next sentence was increased by 2 dB. If the response was incorrect the noise level was decreased by 2 dB. This adaptive procedure targets the required to perceive 50% of the sentences correctly. The SRT was the average signal- to- noise ratio (SNR) from sentence 5–21. The 21st sentence was not presented but its SNR was determined by the response to the 20th sentence.

*Stationary noise* consisted of the speech shaped noises developed by Nilsson et al. (1994) and Hällgren et al. (2006). The spectrum of the noise was shaped according to the long-term average spectrum of the speech material of the corresponding set (same procedure for Swedish and English).

*Fluctuating noise* was constructed by modulating the speech-shaped noise of the target speech by the envelope of the two-talker babbles (see below). Theses envelopes were calculated by extracting the instantaneous amplitude of the babble which was low-pass filtered with a cut-off frequency of 32 Hz (for details see Agus et al., 2009). Two modulated noises were used; one was matched spectrally to the Swedish target speech and temporally to the Swedish babble and one was matched spectrally to the English target speech and temporally to the English babble.

#### Two-talker babble maskers

The Swedish two-talker babble consisted of speech by one native Swedish female and one native Swedish male. They were reading from articles in Swedish newspapers. The English babble also contained speech from a male and a female speaker. The female was a native American English speaker and the male was a native British English speaker. They were reading from articles in English/American newspapers. Both the English and the Swedish two-talker babble maskers were created by mixing the sound-tracks of the male and female speakers.

The data were collected in one session of ~3, 5 h. The session started with the audiometric test, followed by the experimental test and was finished after the cognitive tests. The order of the cognitive tests was counterbalanced. Each participant was tested individually and received oral instructions prior to each test. The audiometric test and the experimental test took place in a sound-attenuated booth and the cognitive tests in a nearby room. Auditory stimuli were presented over headphones (Sennheiser HD600).

#### Tests of relevant capacities

***Reading Span.*** The Reading Span test is a measure of working memory capacity (Daneman and Carpenter, 1980; Rönnberg et al., 1989). The participants were presented three-to-five word sentences, word by word on a screen. The sentences were either irrational (The pear went out) or made sense (The pupil arrived late). After every sentence, participants were asked from the screen to judge whether the sentence made sense or not. They pressed a button; yes or no, according to their answer. After a sequence of three, four or five sentences, the experimenter asked the participant to repeat either the first or the last words in the previous sequence of sentences. The total number of recalled words was scored (maximum score = 23).

***Raven.*** The Raven standard progressive matrices (Carpenter et al., 1990) is a multiple choice measure of non-verbal reasoning. The test was used to assess the ability to – among given alternatives – identify which pattern completes a larger pattern. It includes sets A–E, and every set contains 12 items. Within each set, the difficulty of the matrices increases and so do the subsets, so each of the five subsets is progressively more complex than the previous set. Participants performed set B–D. Responses were scored according to the total number of correct items (maximum score = 36).

***English test.*** The English proficiency test (http://www.nafs.gu.se/digitalAssets/1193/1193558_last_exp.pdf) is a standardized, national test basically developed for students at the gymnasium level. The test assessed the participants’ comprehension in English. The test consists of a text and two parts of questions. The first part includes open questions about the text to answer with the participants own words. The second part consists of sentences with one word in each sentence printed in bold. That bold-printed word should be explained with a synonym at the open end of each sentence in order to make the sentence complete (maximum score = 12).

### STATISTICAL ANALYSES

First, the descriptive statistics of the SRTs in Swedish and English in the four noise conditions were calculated. Then, we performed a repeated measures analysis of variance (ANOVA) on the SRTs with *language* (Swedish, English) and *masker-type* (stationary, fluctuating, two-talker babble Swedish, two-talker babble English) as within-subject factors. We also assessed the descriptive statistics of the English proficiency test, Raven and Reading Span in Swedish and English. Furthermore we assessed the associations (Spearman correlation coefficients) between Reading Span in English and Swedish, English proficiency and Raven on the one hand and the SRTs in Swedish and English on the other hand. Finally, we performed a repeated measures ANOVA with within-participant factors *language* and *masker-type* and with the additional between-participant factor *English proficiency*.

## RESULTS

The means and standard deviations of the SRTs in the eight different conditions are shown in **Table 1**. When comparing the SRTs in each condition between target languages, the SRTs in English are consistently higher. This implies more difficulties when the target speech is in the non-native language. 

**Table 1:** Means and SDs (between parentheses) of the SRTs in Swedish and English.

	SRT-stat	SRT-fluc	SRT-BS	SRT-BE
Swedish target speech	-4.6 (0.9)	-4.1 (1.5)	-0.8 (2.0)	-3.4 (2.2)
English target speech	-0.5 (2.6)	-1.5 (3.5)	2.3 (3.2)	2.6 (3.4)

*SRT = Speech Reception Threshold, stat = stationary noise, fluc = fluctuating noise, BS = Babble Swedish, BE = Babble English.*

A repeated-measures ANOVA, testing the main and interaction effects of *language* (Swedish and English) and *masker type* (stationary noise, fluctuating noise, babble Swedish, babble English) demonstrated main effects of both factors on the SRT thresholds: *Language*: *F*(1,22) = 54.8; *p* < 0.001 and *masker type*: *F*(3,66) = 48.6; *p* < 0.001, respectively. This means that the SRTs were better when the target language was Swedish (mean = -3.17) than English (mean = 0.63). The babble Swedish masker was the most interfering, followed by the English masker. Stationary and fluctuating noises were the least interfering maskers. The interaction between language and masker type was significant, *F*(3,66) = 9.58; *p* < 0.001. The post hoc *t*-tests (Bonferroni adjusted for multiple comparisons at the 0.05 level) showed that when the target was Swedish, the babble Swedish was interfering the most, compared to English babble, stationary noise and fluctuating noise [*t*(66) = 5.26, 7.77, and 6.74, respectively; *p* < 0.001]. When the target was English, the babble Swedish and the babble English were more interfering than the stationary noise [*t*(66) = 5.79, 6.51, respectively; *p* < 0.001] and the fluctuating noise [*t*(66) = 7.77, 8.49, respectively; *p* < 0.001]. This shows that when the target was English, the informational maskers were more interfering than the energetic maskers.

**Table 2** shows the descriptive statistics of the performances on the English proficiency, Raven, Reading Span Swedish and Reading Span English tests.

**Table 2:** Means and SDs in verbal and non-verbal tests.

Eng proficiency	8.2 (3.2)
Raven	31.8 (2.7)
Swe RSpan	14.7 (2.9)
Eng RSpan	11.2 (3.4)

*RSpan = Reading Span, Swe = Swedish, Eng = English.*

**Table 2** suggests that the mean English Reading Span was lower than the mean Swedish Reading Span. A paired-sample *t*-test showed that English Reading Span was indeed lower than Swedish Reading Span *t*(22) = 7.1; *p* < 0.001.

The significantly lower English Reading Span performance is likely due to lower ability in the English language and not in working memory *per se*, as Swedish Reading Span is probably less influenced by individual differences in Swedish language ability.

### CORRELATION ANALYSIS

We computed Spearman correlation coefficients between the measures of cognitive ability, English proficiency, Raven, Reading Span Swedish, and Reading Span English and the SRTs in the eight conditions. The results are presented in **Table 3**.

**Table 3:** Spearman correlation between several test outcomes/abilities and the SRTs in the eight conditions.

	Swedish target speech				English target speech			
	Stat	Fluc	BS	BE	Stat	Fluc	BS	BE
Eng proficiency	0.01	0.38	-0.06	0.31	-0.60**	-0.48*	-0.65**	-0.51*
Swe Reading Span	0.42*	0.30	0.13	0.37	-0.29	-0.26	-0.35	-0.35
Eng Reading Span	0.00	0.20	-0.42*	-0.11	-0.42*	-0.40	-0.44*	-0.58**
Raven	0.26	0.05	-0.08	0.23	-0.24	-0.11	-0.08	-0.22

*p < 0.05; **p < 0.001. Stat = Stationary noise, Fluc = Fluctuating noise, BS = Babble Swedish, BE = Babble English, Eng = English, Swe = Swedish.

**Table 3** shows no significant associations between Raven and any of the SRTs. Swedish Reading Span was associated with one of the SRTs and English Reading Span was associated with four SRTs. The significant associations are probably based on individual differences in English proficiency rather than performance in working memory. English proficiency was associated with lower (better) SRTs when the target language was English, but not when it was Swedish, indicating that English proficiency was especially important when speech perception concerned the English language. Age was significantly correlated to English proficiency and the SRTs. This may suggest that English proficiency is related to age. To gain more insight in this issue, we assessed the relations among the variables when age was controlled for. For this, we performed a partial correlation analysis. However, the results were similar to those previously, when age was not controlled for. Therefore, we conclude that the effect of English proficiency was not related to age.

**FIGURE 1:**
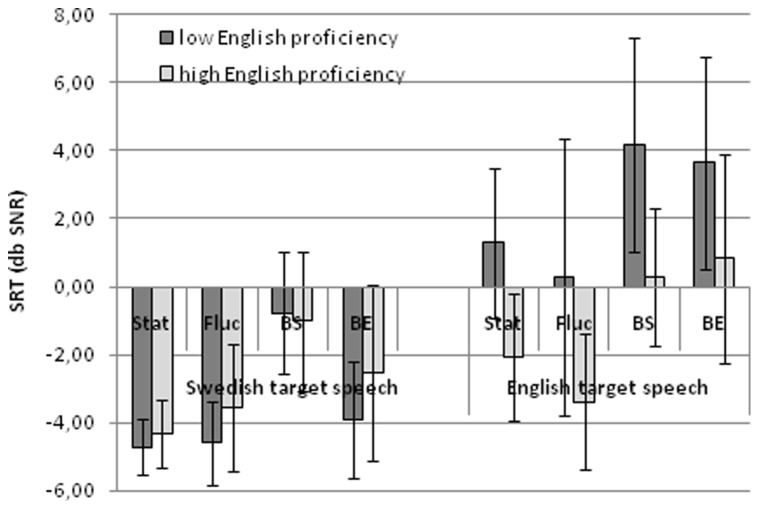
**The mean SRTs for the high and low English proficiency group in each of the eight conditions.** Error bars reflect standard deviations

In order to further assess the relevance of individual differences in English proficiency for English target speech, we divided the group into two subgroups based on English proficiency. Therefore, we first calculated the median English proficiency score which was 9. Participants with English proficiency below 9 were grouped into the “low English proficiency group” (*n* = 11, mean = 5.36, SD = 2.04) and participants with English proficiency above 9 were in the “high English proficiency group” (*n* = 10, mean = 11.05, SD = 0.90). See **Figure 1**. Two participants with the median score of 9 were excluded from further analyses.

To examine how the frequency with which the participants use English affected English proficiency, we checked the different frequency variables against the high (H) and low (L) English proficiency groups. The results were as follows; “Daily (3 H, 1 L),” “Every week (5 H),” “Every month (2 L),” “Every year (2 H, 3 L),” “Holidays (4 L),” and “Never (1 L).” The results generally indicate that daily or weekly use of English, probably improve performance in this non-native language, however we cannot be conclusive as regards causality.

We again performed a repeated measures ANOVA with the within-participant factors *language* and *masker type* but with the additional between-participant factor *English proficiency group* (high and low proficiency).

The main effects of language and masker type were similar as described above (results of the first ANOVA analysis). The main effect of English proficiency was not significant *F*(1,19) = 3.68; *p* = 0.07. The ANOVA showed an interaction effect between *language* and *English proficiency group*: *F*(1,19) = 30.3; *p* < 0.001. The *post hoc t*-tests indicated that the high English proficiency group performed better than the low proficiency group when the target was English, *t*(19) = 3.40; *p* < 0.001 but not Swedish, *t*(19) = 1.14; *p* = 0.13.

There was no interaction effect between *English proficiency group* and *masker type F*(3,57) = 1.22; *p* = 0.312

## DISCUSSION

This study examined how English proficiency affected native and non-native speech perception under energetic and informational maskers. The assessment of external as well as internal, listener-related factors allowed us to examine which variables explained intra- and interindividual differences in native and non-native speech perception performance.

The main result of the study is that the individuals’ English proficiency level considerably affects speech recognition in noise. This finding was revealed by the new approach with focus on proficiency and the extended design of the study. For English (non-native) target speech, the SRTs were substantially lower (better) for high English proficiency-listeners as compared to listeners with lower English proficiency levels. This effect of English language proficiency did not emerge for Swedish target speech. As expected, the listeners achieved lower (better) SRTs when the target speech was native. We also predicted that the informational maskers should interfere more with perception than the energetic maskers. Indeed, in English (non-native) as target speech, the performance was worse for the informational maskers (i.e., the two-talker babbles) compared to the energetic maskers (fluctuating noise and stationary noise). In Swedish (native) as target the babble Swedish was interfering more than the English babble, stationary noise and fluctuating noise. The interference from language maskers replicates previous work (Van Engen and Bradlow, 2007; Calandruccio et al., 2010). These studies have found that the language of the maskers might interfere with the intelligibility of the target speech. The difficulties may be explained in different ways. It is possible that the difficulties are (a) due to the intelligible words in the masker (Hoen et al., 2007; Van Engen and Bradlow, 2007), or it is possible that the difficulties are (b) due to linguistic similarity between the target and the masker (Brouwer et al., 2012), (c) or both. The current study suggests that the effect of target language and the intelligibility of the masker speech are mediated by the individuals’ ability to perceive non-native speech, as non-native proficiency interacted with masker type and target language. For Swedish as target speech, both groups performed better in the English babble masker compared to the Swedish babble masker. However, the low proficiency group received the largest release of masking from the English babble, possibly due to their low proficiency in English and thus reduced understanding of the masker speech, which made it easier to suppress.

An interesting fact is that the SRTs of the low English proficiency group for English target speech with both English and Swedish speech maskers were similar to their SRTs in fluctuating noise. This indicates that when it is relatively difficult to identify the interfering speech, the interference is similar to that imposed by energetic noise. If the language of the speech is unfamiliar, there remains nothing but a fluctuating masker similar to an energetic noise masker. This shows the relevance of including both speech and noise maskers.

The interaction effect between English proficiency and language, indicated that for English target speech, the low proficiency group performed significantly worse in the babble maskers compared to the high proficiency group. Also, the low proficiency group performed at a similar poor level in both English and Swedish babble maskers (see **Figure 1**). Comparable performances in both the native and non-native babble maskers were also observed for the highly proficient group, but at considerably lower (better) SNRs (see **Figure 1**).

We did not observe a main effect of language proficiency subgroup. This indicates that the influence of language proficiency depends on masker and target speech characteristics, and does not in itself constitute a general superiority of the high-proficiency group to understand speech in general. The present results demonstrate the importance of taking into account language proficiency when assessing speech perception performance.

The current finding that listeners in non-native target conditions perform similarly for native and non-native speech maskers is consistent with the study of Garcia Lecumberri and Cooke (2006) but contrasts with Van Engen (2010) and Brouwer et al. (2012). In Van Engen (2010), when target speech was English, the non-native Mandarin-speaking participants had larger difficulties in the English babble than in the Mandarin babble. In Brouwer et al. (2012), non-native Dutch listeners perceived a non-native target (English) better when the masker speech was native (Dutch) as opposed to non-native masker. The task (sentence-recognition) and the masker speech (two-talker babble) were comparable in the current and previous studies. Therefore, the differences in the results can possibly be attributed to different characteristics of the participants in the studies. First, the listeners’ proficiency in the non-native language differed between the studies. In Van Engen (2010), the Mandarin-speaking participants had previously attained a university-based general proficiency test. Additionally, before they took part in the study, they rated their proficiency on a scale from 0 to 10. The mean rating of 6 indicates “slightly more than adequate” (Van Engen, 2010). The non-native listeners in Brouwer et al. (2012) were assumed to have relatively high non-native language proficiency. In the present study, we objectively assessed individual differences in non-native language proficiency and we observed that the listeners varied considerably in this ability. The present study underscores the relevance of proficiency in a second language for speech perception. Therefore, differences in the proficiency level of the listeners included in our study versus previous studies may have played a role. Secondly, the age-range of the listeners was wider in the current as compared to previous studies. This may have resulted in more interindividual variance in the SRTs, and poorer SRTs overall.

In Cooke et al. (2010), eight listener groups with different native languages identified consonants in a competing speech masker, speech-shaped noise and speech-modulated noise. Most of the listener groups performed relatively poor in the conditions with competing speech. The impact of the masker speech was associated with self-rated non-native language proficiency. This indicates that language proficiency affects the ability to ignore competing speech. Additionally, the adverse impact of a competing speaker was smaller for listeners who performed well overall. To resist or inhibit competing speech is an ability that seems to go with being bilingual – in the sense of being raised as a bilingual. According to Bialystok (2001) and Bialystok et al. (2004) bilingualism improves inhibition control. The constant exercise in suppressing either language develops the ability to ignore distracters, not only for other languages, but also other types of irrelevant stimuli. Although speculative, in the present study for English as target speech, we consistently observed that the subgroup with high non-native language proficiency was better to inhibit each type of masker signal as compared to the subgroup with low English proficiency.

For the low proficiency group, the process of decoding the non-native speech in noisy conditions was probably more effortful than for the high proficiency group. In the present study, we observed a relation between English Reading Span and the four SRTs with English target speech. This association is probably based on the fact that in each of these tests, the target language was English. This is supported by the fact that the relationships between Swedish Reading Span test and the English SRTs were somewhat weaker. We suggest that the importance of both English proficiency and working memory capacity for performing the English Reading Span test makes it a slightly better predictor of the English SRTs than the Swedish Reading Span test for either the Swedish or English SRTs. Like the English Reading Span test, the Swedish test may have been sensitive to differences in Swedish proficiency. However, we assume that in this group of Swedish native speakers, this has not played a role. Better performance on the Swedish Reading Span test was however associated with worse SRTs in stationary noise for Swedish target speech. We did not expect this relationship. Inspection of this association indicated that the relation was mainly driven by the result of one listener with a relatively poor SRT in stationary noise and good Reading Span performance. Raven was not associated with any of the SRTs, presumably due to the non-verbal nature of the test. However, the results need to be interpreted cautiously due to the large number of correlations and the small sample size.

The present study focused on different aspects that might influence speech perception in a non-native language under challenging conditions. For a better understanding of this complex process both external and internal factors were taken into account. Internal or listener-related factors that we assessed included cognitive abilities like working memory and proficiency in a non-native language. The merits of this study, compared to previous ones are: (a) the focus of proficiency in the non-native language (b) application of a standardized test of English proficiency measured on a continuous, objective scale (c) a wide age-range (d) the application of a within-subjects design while using e) SRT-tests for four different masker types, with each participant being tested in both target languages.

This study attests to the importance of individual, objective assessment of non-native language proficiency as it affects speech perception in a non-native language. Previous studies have also indicated that working memory predicts speech perception in challenging conditions (e.g., Akeroyd, 2008; Rönnberg et al., 2008, 2010). However, the current data do not indicate strong associations between working memory (here measured with Reading Span tests) and speech perception in a non-native language. More research is required that explores these association in larger sample sizes.

In conclusion, we observed that non-native language proficiency to a large extent influences speech perception in noise. The study has provided evidence in support of the prediction that informational maskers affected speech intelligibility more than energetic noise maskers. The SRTs were lower (better) when the target language was native compared to target speech in the non-native language. We observed interactions between the target language, the language of the interfering speech and proficiency in the non-native language. This finding suggests that in difficult listening conditions including non-native target speech that is masked by interfering speech, high proficiency in the non-native language is an apparent advantage.

## Conflict of Interest Statement

The authors declare that the research was conducted in the absence of any commercial or financial relationships that could be construed as a potential conflict of interest.
